# Four-dimensional measurement of the displacement of metal clips or postoperative surgical staples during 320-multislice computed tomography scanning of gastric cancer

**DOI:** 10.1186/1748-717X-7-137

**Published:** 2012-08-10

**Authors:** Hideomi Yamashita, Kae Okuma, Wataru Takahashi, Akira Sakumi, Akihiro Haga, Kenji Ino, Masaaki Akahane, Kuni Ohtomo, Keiichi Nakagawa

**Affiliations:** 1 Departments of Radiology, University of Tokyo Hospital, Tokyo, Japan

**Keywords:** Intra-fraction motion, Four-dimension computed tomography, Gastric cancer, Internal margin, Respiratory motion

## Abstract

**Purpose:**

To investigate the respiratory motion of metal clips or surgical staples placed in the gastric wall for planning of radiation therapy in gastric cancer patients.

**Methods:**

This study examined 15 metal markers in the gastric walls of 12 patients with gastric cancer treated with external-beam photon RT. Motion assessment was analyzed in 41 respiratory phases covering 20 s acquired with computed tomography (CT) in the RT position using 320-multislice CT. The intra-fraction displacement was assessed in the cranio-caudal (CC), antero-posterior (AP), and right-left (RL) directions.

**Results:**

Motion in the CC direction showed a very strong correlation (R^2^ > 0.7) with the respiratory curve in all 15 markers. The mean (+/− SD) intra-fractional gastric motion (maximum range of displacement) was 12.5 (+/− 3.4) mm in the CC, 8.3 (+/− 2.2) mm in the AP, and 5.5 (+/− 3.0) mm in the RL direction. No significant differences in magnitude of motion were detected in the following: a) among the upper (n = 6), middle (n = 4), and lower (n = 5) stomach regions; b) between metal clips (n = 5) and surgical staples (n = 10); and c) between full (n = 9) and empty (n = 6) stomachs.

**Conclusions:**

Motion in primary gastric tumor was evaluated with 320-multislice CT. According to this study, the 95th percentile values from the cumulative distributions of the RL, AP, and CC direction were 6.3 mm, 9.0 mm, and 13.6 mm, respectively.

## Introduction

Inter-fraction and intra-fraction motion of critical structures are a significant concern when patients undergo intensity-modulated radiotherapy (IMRT). Improper dose modulation can be a result of anatomical motion. Over-dosage to normal tissues can result in toxicity, whereas under-dosage to tumors can lead to tumor progression. Image-guided radiotherapy (IGRT) has been used in an attempt to minimize the impact of anatomic motion [1].

Standard treatment for gastric cancer in Europe and the US at present is neoadjuvant chemotherapy followed by surgery (minimum resection margin 4 cm, which will lead to total gastrectomy in many cases). In more advanced stages or R1- resections, surgery is followed by chemoradiation (CRT). The motion in primary esophageal tumors and breast cancers evaluated with 320-row multi-slice CT (320 MSCT) has been reported previously, respectively [2,3]. 4D-CT enables gastric motion to be tracked over the entire length of the organ and during all phases of the respiratory cycle. The present study used continuous data acquisition and not gating. The connection with breathing motion is only facilitated by measuring the partial lung volume during post-processing. The present study analyzed the 3D movement over time of 15 metal markers in the gastric wall using 320 MSCT. This study of 12 patients represents the largest analysis heretofore conducted of intra-fraction gastric movement during free respiration.

## Methods and materials

### Patient information

Intra-fraction motion of the stomach was analyzed in 12 patients with gastric cancer who were treated with adjuvant or preoperative radiotherapy between June 2010 and February 2011 at University of Tokyo Hospital, Tokyo, Japan. The clinical and demographic data of the 12 patients (all males) are shown in Table 1. All patients suffered from histopathologically confirmed adenocarcinoma of the stomach. The median age was 74 years (range, 50–87 years).

**Table 1:** Summary of patient characteristics

**Patient**	**Age(years)**	**State**	**MArkrer**	**Location of marker**	**Stomach filling**	**TNM**
1	81	Preoperative	Metal clip	Upper	Full	cT2NMO
2	58	Postoperative	Surgical staple	Middle	Full	pT3N1MO
3	75	Preoperative	Metal clip	Low	Slight	cT3N3MO
4	62	Postoperative	Surgical staple	Low & middle & upper	Slight	pT3N1MO
5	87	Postoperative	Surgical staple	Low	Full	pT3N2MO
6	74	Postoperative	Surgical staple	Upper	Full	pT4N3MO
7	81	Preoperative	Metal clip	Middle	Empty	cT4N1MO
8	73	Preoperative	Metal clip	Upper	Empty	cT3N3MO
9	50	Preoperative	Metal clip	Upper	Full	cT3N2MO
10	78	Postoperative	Surgical staple	Upper	Full	pT3N1MO
11	71	Postoperative	Surgical staple	Low	Full	pT4N1MO
12	73	Postoperative	Surgical staple	Low &middle	Full	pT4N3MO

Written informed consent was obtained from all patients before initiating treatment. This study was approved by the institutional review board (IRB) of Tokyo University (No. 2613). The consent details were: participation in this study was voluntary, that the radiation exposure dose received by the participants was approximately 40–50 mSv, and that the institute would bear all costs of this CT study, in addition to managing and defining the outline, purpose, and method of this independent study and assuring privacy protection. This study was subsidized by Grants-in-Aid for Scientific Research (KAKENHI) in 2010: Grant-in-Aid for Young Scientists (B).

### Surgical procedure

There were 5 preoperative and 7 postoperative patients. The operative procedure was limited to only partial gastrectomy for 8 patients. Patients who received total gastrectomy were excluded from the study because there was no stomach to evaluate motion. The 320 MSCT was performed after placement of a metal clip (EZ endoclip, HX-610-090; Olympus, Tokyo, Japan) in the normal gastric wall near the primary tumor for 5 preoperative patients or after placement of surgical staples (Multifire endo GIA, ENDO TA^TM^ 30, COVIDIEN, Tokyo, Japan) during partial gastrectomy for 7 patients. When fully closed, the clip resembles a cylindrical structure. The length is approximately 10 mm, and the diameter is approximately 2 mm. Each tumor had one or two metal markers, and 15 markers in 12 patients were analyzed. The preoperative patients underwent endoscopy for marker placement. The marker was placed adjacent to the primary tumor in order to show the gross tumor volume clearly. Two clips in the both cranial and caudal end of the primary tumor were placed in three patients out of them. The stomach was divided into three segments along the longitudinal axis (from gastro-esophageal junction to pyloric canal): upper (U), middle (M) and lower (L) thirds. Out of the 5 preoperative patients, 4 patients can receive radical total gastrectomy after preoperative CRT, but one patient cannot receive radical surgery due to progression of the gastric cancer during preoperative CRT.

### Imaging protocol

A 320 detector lines volumetric CT scanner (320-slice Aquilion ONE; Toshiba Medical Systems, Otawara, Tokyo) was used to scan the metal gastric markers for 20 s [4]. Images were acquired in volume mode (16 cm z coverage per rotation). The scan slice thickness (= detector row interval) and image slice thickness and reconstruction interval every 41 phases was 0.5 mm, 1.0 mm, and 1.0 mm, respectively. We used dynamic volume 4D-CT not respiratory correlated 4DCT for motion assessment.

The time for a single rotation was 0.5 s. One image per 0.5 s rotation for 20 s leads to the 41 phases. The scan settings were 120 kVp and 50 mAs, reconstruction filter was used, voxel size was 512 x 512 (field of view = 500 mm), and scan mode was static cine-mode, not helical.

The flat board (CABMO21A) was used in place of a body mat, to flatten the superior surface of the patient table. Thus, imaging could be performed in the same position as RT. The scans were all performed at 16:30 before dinner without any dietary restriction.

### Image analysis

**Figure 1:**
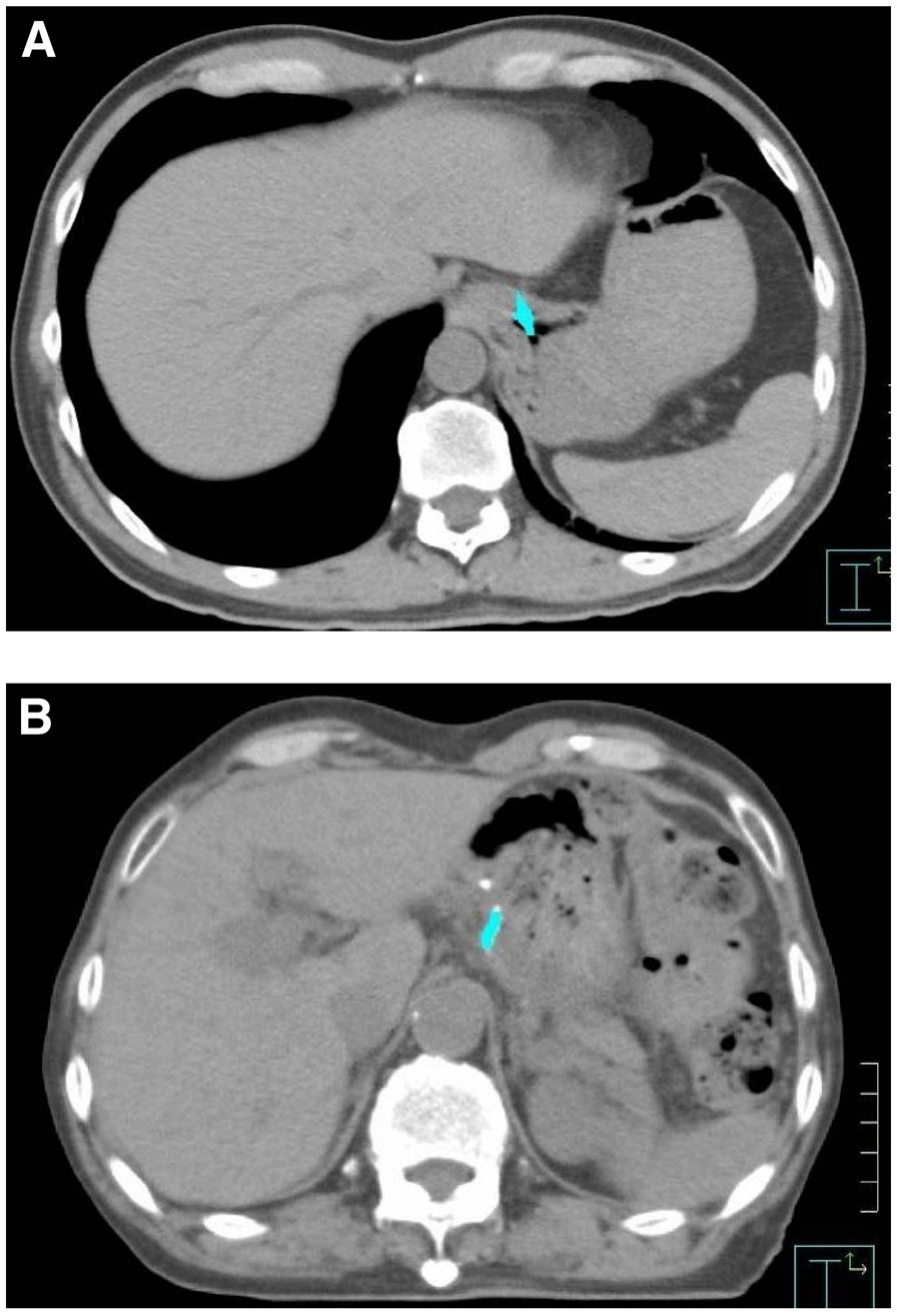
**Contouring of metal marker and center of gravity of each metal marker, performed on a Pinnacle**^**3**^** workstation.** (**A**) Metal clip. (**B**) Surgical staple.

Fifteen metal clips or surgical staples and bilateral lung delineation was performed for each phase and each patient (total 41 sets for each metal clip) on a Pinnacle^3^ treatment-planning workstation. The delineation was performed semi-automatically. The lower auto-contour threshold was set at 400 Hounsfield units for the metal clips and −200 Hounsfield units for the lung. The coordinates (x, y, and z) of the center of gravity of each metal clip were calculated automatically on a Pinnacle^3^ workstation (Figure 1). Positive directions were right to left on the x-axis, from posterior to anterior on the y-axis, and from superior to inferior on the z-axis.

**Figure 2:**
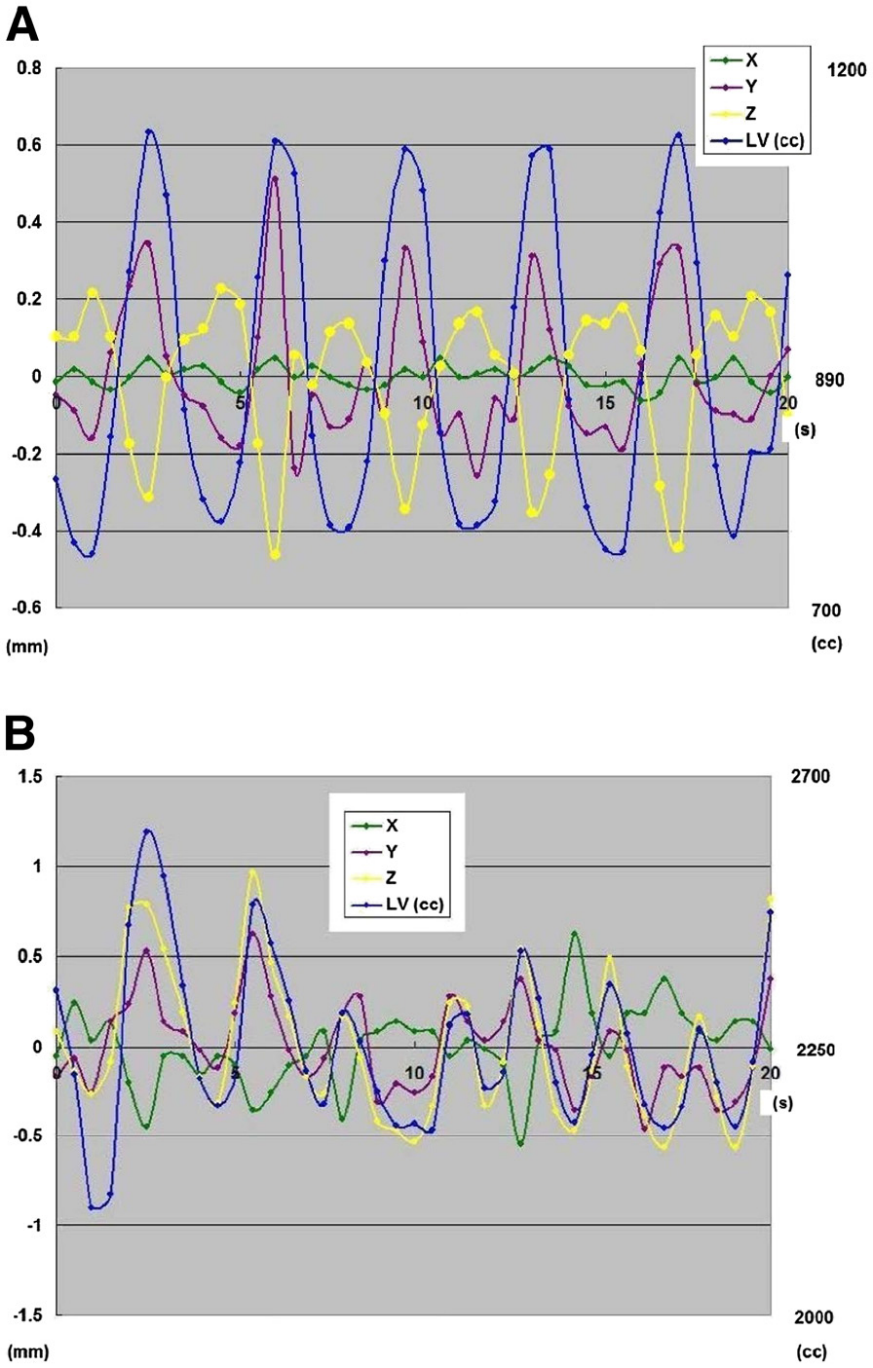
**Centroid trajectories of metal clips in all 41 phases for 20 s of the four-dimensional CT scans.** (**A**) Patient 6. (**B**) Patient 1. RL = right–left; AP = anterior–posterior; CC = cranio–caudal.

The respiratory phase was assessed by contouring the partial lungs semi-automatically on a Pinnacle^3^ treatment-planning workstation (Philips Healthcare, Andover, MA; ADAC, Milpitas, CA), thereby determining the change of partial lung volume (right vertical axis in the Figure 2). The position (z-coordinate) of dome of the diaphragm was measured for each respiratory phase that was measurable in 7 patients (Cases No. 5–11) (because in this 7 patients the dome was fully included within CT scan range in all 41 phases) in order to confirm the correlation with the change of partial lung volume (cc) only within CT scan range.

When the contents of the stomach could be recognized clearly by 320MSCT, it was defined as a full stomach. Otherwise, it was defined as an empty or almost empty stomach.

### Statistical analysis

We calculated *R*^*2*^ values (decision coefficient, i.e., the square of correlation coefficient) to assess the possible correlation between metal displacements and bilateral lung volume in 41 phases only within the scan range of 16 cm in the CC direction, not full length of the lung. We did not need the zero value of the breathing curves. The mean value and SD were calculated for all 15 *R*^*2*^ values each metal marker in all CC, RL, and AP directions. We also calculated the *p* value of *R*^*2*^ values using non-parametric Spearman test.

Secondly, we calculated the mean and median value and SD for 15 values of difference between the two extreme values each metal marker in all CC, RL, and AP directions.

Finally, from these displacement data, the 95th percentile displacements, which were given the percentiles of 615 pooled data (41 values/case x 15 metals) of difference between every 41 point values and minimum value of each metal, were determined in all CC, RL, and AP directions.

## Results

In the procedure of marker insertion by endoscope for 5 preoperative patients, no symptomatic complications were experienced. The locations of the 15 metal markers are shown in Table 1. There were 6, 4, and 5 markers in the upper, middle, and lower locations, respectively. The stomach was full in 8 patients and empty (including almost empty) in 4 patients, which was judged by its appearance on CT (Table 1).

Motion in the CC direction showed a very strong correlation (R^2^ > 0.7) with the respiratory curve in all 15 markers (mean +/− SD = 0.86 +/− 0.09, range; 0.74-0.97), regardless of upper, middle, or lower stomach locations (Table 2). For the RL (0.52 +/− 0.27, 0.10-0.81) and AP (0.68 +/− 0.24, 0.20-0.95) directions with 11 markers (73 %) and 14 markers (93 %), respectively, also showed a strong correlation (R^2^ > 0.4) with the respiratory curve. The changes of partial lung volume for each respiratory phase showed a very strong correlation (R^2^ = 0.90, 0.89, 0.96, 0.98, 0.98, and 0.99, and all p < 0.01) in 6 of 7 patients with the position (z-coordinate) of the diaphragmatic top with one exception (R^2^ = 0.46, *p* = 0.047).

**Table 2:** Amplitudes of metal markers and correlation with the respiratory curve

	**Max-min (mm)**			**R2 with lung volume**		
	**RL**	**AP**	**CC**	**RL**	**AP**	**CC**
Mean (σ)	5.5	8.3	12.5	0.52	0.68	0.86
Median	5.7	7.8	13.6	0.60	0.72	0.84
SD	3.0	2.2	3.4	0.27	0.24	0.09
Max	11.7	13.0	16.3	0.81	0.95	0.97
Min	1.1	5.1	6.9	0.10	0.20	0.74

*Abbreviations*: *RL* = right-left; *AP* = anterior-posterior; *CC* = cranio-caudal.

The respiratory curve for 20 s showed a regular periodic motion in 11 patients (Figure 2-A) and a slightly irregular one in the last patient, who was 81 years old (Figure 2-B). The respiration frequency during 20 s ranged from 4.0 cycles (i.e., 5.0 s per cycle) to 7.0 cycles (i.e., 2.9 s per cycle), with median values of 5.3 cycles and 3.8 s per cycle. Three cases (25 %) would be chosen a different planning volume if only one breathing cycle had been used for planning.

The mean amplitudes of the marker from the min to the max values (= maximum range of displacement) are shown in Table 2. The mean +/− SD amplitudes of the marker movements were 5.5 +/− 3.0 mm (max, 11.7 mm), 8.3 +/− 2.2 mm (max, 13.0 mm), and 12.5 +/− 3.4 mm (max, 16.3 mm) in the RL, AP, and CC directions, respectively. In other words, the intra-fractional gastric motions in the CC direction ranged from 6.9 mm to 16.3 mm (mean; 12.5 mm). There was neither significant difference in magnitude of motion between upper (6 clips), middle (4 clips), and lower (5 clips) stomach regions, nor between metal clips (5 clips) and surgical staples (10 clips), nor between full (n = 8) and empty or almost empty (n = 4) state in the stomach for the remaining patients (Table 3).

**Table 3:** Amplitudes of the marker movements by each specification

		**max-min (mm)**					
	**N**	**RL**		**AP**		**CC**	
		**Mean**	**SD**	**Mean**	**SD**	**Mean**	**SD**
Location							
Upper	6	5.3	4.1	8.8	1.3	11.5	3.9
Middle	4	3.7	1.7	6.0	1.2	13.6	3.8
Lower	5	6.9	0.5	9.2	3.3	13.5	3.0
Gastric contents							
Full	9	5.6	3.6	9.0	2.2	12.8	3.9
Empty or almost empty	6	5.2	1.5	6.7	1.4	11.9	2.4
Marker							
Metal clip	5	6.8	3.5	8.3	2.5	13.4	2.0
Surgical staple	10	4.6	2.6	8.4	2.3	11.9	4.2

The 95th percentile values from the cumulative distributions were used to define minimum margins to account for gross tumor volume motion during treatment planning. The values of the RL, AP, and CC direction were 6.3 mm, 9.0 mm, and 13.6 mm, respectively.

## Discussion

In this study, metal markers in the stomach were evaluated on the 320 MSCT. The literature describing gastric tumor motion is limited. Because the motions of 15 metal markers were analyzed and the contouring was performed semi-automatically using the threshold CT value, the human error of contouring appears to be very small as compared with contouring the outline of lung or pancreatic tumor or gastric wall by freehand. Moreover, because the continuous cine-mode shooting for 20 s under free breathing was performed over 16 cm in the CC direction using the 320 MSCT, no respiratory monitoring system like the Real-Time Positional Management (RPM) (Varian Medical Systems, Palo Alto, CA) or the AZ-733 V system (Anzai Medical, Tokyo, Japan) was used, and the respiratory curves were made using the change of partial lung volumes for each respiratory phase. The lung volume change was used as the reference value for the breathing state in this study. However it could not be used for real time gating because the processing time was longer than the diaphragmatic position, thoracic circumference, or y-position of the upper abdominal wall, which are more realistic parameters for tracking.

Respiratory, cardiac motion, and bowel peristalsis are the main contributors to intra-fractional organ motion. Many groups have evaluated intra-fractional motions of the liver, diaphragm, kidney, pancreas, lung tumors, and prostate [1,5-11]. In a previous report, voluntary breath hold inspiration and expiration CT scans were used to assess the gastric stability and estimate the breathing motion [12]. However, to the best of our knowledge, this is the first study that systematically evaluated the intra-fractional gastric motion of metal markers using 4D-CT.

With the more widespread availability and the increasing use of sophisticated treatment techniques such as IMRT and IGRT, adequate safety margins in gastric cancer need to be addressed. While RT for gastric cancer has traditionally involved quite large volumes – Smalley and co-workers [13] have given an impressive example – toxicity and hence treatment volumes should be reduced if at all possible. We investigated the respiratory motion of metal clips or surgical staples placed in the gastric wall in gastric cancer patients. The investigations in this study were selected who received partial gastrectomy for the postoperative cases and who agreed to receive 20 s continuous scanning by the 320 MSCT for all cases.

Because there was no significant difference in magnitude of motion between pre- and post-operative patients, there were no differences attributable to fibrosis post surgery in this study.

According to Watanabe *et al.*[14], the mean intra-fractional gastric motions for 11 gastric lymphomas using fluoroscopic examinations at the time of the simulation were 11.7, 11.0, 6.5, 3.4, 7.1, 6.6 mm for the superior, inferior, right, left, ventral and dorsal points, respectively, which were significantly different between each point. According to Wysocka *et al.*[15], the median respiratory amplitudes for 22 postoperative gastric cancers using serial study CT scans in free breathing, voluntary inhale and exhale were 1.7, 4.8, and 14 mm in the RL, AP, and CC direction, respectively. These values in our study were generally equal to those in reports mentioned earlier [12,14]. The motions were not different among upper, middle, or lower stomach, between metal clips or surgical staples, and between full or empty/slight stomach states. In addition to the upper stomach, even the middle or lower stomach also showed a strong correlation with the respiratory curve at a high rate. Additionally, the RL or AP directions as well as the CC also showed strong correlations with the respiratory curve at a high rate.

In the free breathing state, respiration is the dominant cause of intra-fraction organ motion during RT. Recently, respiratory sorted CT imaging (4D-CT) [15-18] has been used in order to examine respiratory amplitude. It is likely that the breath hold technique used by Wysocka *et al.*[12] overestimated the deviation during normal quiet breathing. Our result concurred with that of Wysocka *et al.*[12], who reported a CC range from 0 to 60 mm. The scatter of their results was more widespread. Our published data in the present study have reinforced the substantial variations present in breathing motion among patients. Generally, respiratory motion is larger in forced in/expiration than in voluntary breathing. To evaluate the more accurate respiratory motion, we selected the method of voluntary breathing.

We used volumetric cine CT images to clearly visualize gastric movement as 3D trajectory plus time. Our volumetric cine CT sets have high spatiotemporal resolution, resorting of CT images is not necessary, and the 4DCT artifacts as observed in conventional multi-slice CT do not occur [19]. The accuracy of our results is much improved over those obtained using conventional CT. These characteristics highlight the advantages of 320MSCT in volumetric cine imaging over existing multi-slice CTs, although the comparison with the literature suggests little differences to the presented study.

We are aware of certain concerns that may be raised about radiation exposure to patients during the continuous cine-mode shooting for 20 s using the 320 MSCT procedures. Continuous shooting for 20 s may be too long for patients with a regular respiratory cycle (Figures 2-A). However, there was only one patient with an irregular respiratory cycle (Figure 2-B), and in such patients continuous shooting for 20 s was considered necessary to obtain sufficient data about respiratory motion. With our protocol, the dose of radiation exposure from 320 MSCT for 20 s was approximately 40–50 mSv at the skin surface. It may be easy to shorten the scanning time or decrease the current value for dose reduction, although, in return, image quality would be deteriorated and noise would increase. This effort may be possible for the chest but difficult for the abdomen. A secondary concern in this study is the blurring for 0.5 s that occurs in imaging because the time response remains at 0.5 s. In a very quick movement of <0.5 s, an error may occur in the evaluation of the motion. Because the typical breathing period of a patient is approximately 3–5 s, the data were acquired for roughly every 10 % of the respiratory cycle in this study, which is comparable to what typical 4D-CTs acquire using RPM or the Anzai belt. A final concern lies in the resolution of the reading system. Because the resolution is 512 x 512 segments in the transverse, there is a limit to the resolution of reading power. In as much as the motion can be traced to a certain extent in a patient with a broad movement, in a patient with only a small movement, the data will show a poor resolution. The small sample size of 12 patients and 15 metal clips precludes our making firm recommendations regarding adequate ITV margin expansions, although prior studies of esophageal tumor motion had similar sample sizes [12,14]. Additionally, we recognize that the measurements of organ motion based on 4D-CT taken on the day of simulation may not accurately represent the magnitude of motion occurring during subsequent daily RT. Practical considerations make it difficult to obtain multiple 4D scans during the course of treatment. The last limitation is our scope, limited to a single imaging session, thereby making it impossible to study inter-fractional/imaging session aspects of gastric motion that are likely to be considerable.

We used the center of gravity as the procedure for suture lines. Because the number of CT slices which can detect the metal on the axial image was no different in 41 respiratory phases, we consider that there was not deformation of the lines during respiration.

With respect to other published work, we submit that the strengths of this study include the use of normal-breathing 4D-CT scans rather than breath-holding techniques, inclusion of tumors in all gastric locations, and the comparison with a respiratory curve. Our data can perhaps be used to produce guidelines for margins to account for respiratory motion of primary gastric cancers. From this study, we suggest that internal target volume margins need to include adjacent lymph node levels should be 7 mm, 9 mm, and 14 mm in the RL, AP, and CC direction, respectively, in addition to clinical target volume.

## Conclusions

In conclusion, this is the first study that systematically evaluated the intra-fractional gastric motion using 4D-CT. We have demonstrated considerable intra-fractional gastric motion. We have shown that the magnitude of motion can vary from patient to patient.

## Competing interests

The authors declare that they have no competing interest

## Authors’ contribution

HY hit on the idea of this study, carried out this study, and wrote this article. KO and WT carried out the contouring the clips or staples on a Pinnacle3. KI, MA, and KO were supportive to use 320-multislice computed tomography machine and scanned CT. KN compiled this study and this paper. All authors read and approved the final manuscript.
